# *Anaphalis margaritacea* ethanol extract exhibits potent anti-*Trichinella spiralis* activity via mitochondrial dysfunction and host tissue protection

**DOI:** 10.1016/j.ijpddr.2026.100650

**Published:** 2026-05-18

**Authors:** Xiaoyan Yu, Xinyuan Cao, Yuchao Ma, Lixia Dai, Tong Bu, Xiaorong Yang, Zile Gong, Jianing Pang, Yang Huang, Mingze Cao, Zhen Zhu, Xiaolou Miao, Xiaofei Shang

**Affiliations:** a Key Laboratory of New Animal Drug Project, Gansu Province, Key Laboratory of Veterinary Pharmaceutical Development of Ministry of Agriculture, Lanzhou Institute of Husbandry and Pharmaceutical Sciences, Chinese Academy of Agricultural Sciences, Lanzhou, PR China; b School of Life Sciences and Food Engineering, Hebei University of Engineering, Handan, PR China; c People's Hospital of Ningxia Hui Autonomous Region, Ningxia Medical University, Yinchuan, PR China; d College of Pharmacy Gansu University of Chinese Medicine, Lanzhou, Gansu, PR China

**Keywords:** *Trichinella spiralis*, *Anaphalis margaritacea*, Anthelmintic activity, Mitochondrial respiratory chain

## Abstract

Trichinellosis is a food-borne zoonosis caused by *Trichinella spiralis*, for which effective treatment against the muscle larvae stage remains limited. To identify new anti-*T. spiralis* agents from medicinal plants, 30 extracts with reported or presumed antiparasitic relevance were screened in vitro. Among them, the 80% ethanol extract of *Anaphalis margaritacea* (AMEE) showed the strongest larvicidal activity, causing complete muscle larvae mortality within 72 h at 100 μg/mL and exhibiting a clear dose-dependent effect. Preliminary chemical characterization by UPLC-Q-TOF-MS/MS led to the tentative identification of eight major constituents, mainly organic acids and flavonoids. In a murine model of *T. spiralis* infection, oral administration of AMEE reduced muscle larvae burden by up to 59.7% in a dose-dependent manner. AMEE treatment also alleviated muscle fibre damage and inflammatory infiltration in infected muscle, accompanied by reduced levels of IL-6, IL-13, and TGF-β and decreased mRNA expression of *Nqo1* and *Sod2*. To explore the basis of its antiparasitic activity, integrated proteomics, mitochondrial enzyme activity assays, and transmission electron microscopy were performed. AMEE markedly suppressed the activities of mitochondrial respiratory complexes I, II, and IV, reduced complex V activity, and induced mitochondrial swelling and cristae disruption in muscle larvae. These findings show that AMEE exerts significant anti-*T. spiralis* activity in vitro and *in vivo*, with evidence consistent with mitochondrial dysfunction in muscle larvae and attenuation of infection-associated muscle pathology.

## Highlights

• AMEE shows strong in vitro activity against *T. spiralis.*
• AMEE reduces *T. spiralis* muscle larval burden and alleviates muscle pathology in mice.
• AMEE modulates Th2 cytokines, fibrotic markers and oxidative stress in infected muscle.
• AMEE exerts anti-*Trichinella* activity by targeting parasite mitochondria.

## Graphical abstract

**Figure:**
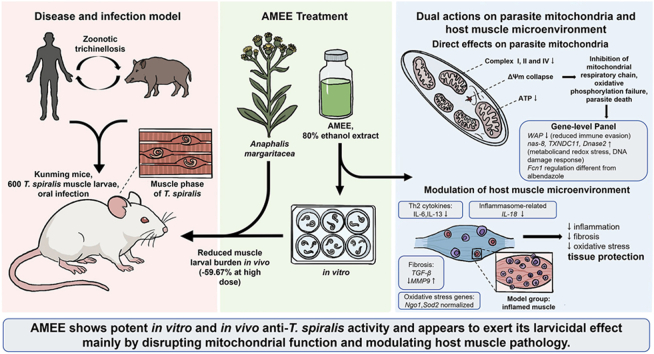

## 1 Introduction

*Trichinella spiralis* (*T. spiralis*) is a significant zoonotic parasitic nematode characterized by a direct life cycle that is completed within a single host, without the need for an intermediate host. Adult worms reside in the small intestinal mucosa, where they release newborn larvae that migrate through the bloodstream to striated muscle tissue, eventually encapsulating into infective larval cysts (Bilska-Zając et al., 2021). The life cycle of *T. spiralis* is distinctly divided into two phases: the intestinal phase and the muscular phase. Human infection commonly results from the consumption of raw or undercooked meat from domestic pigs or wildlife containing infective larvae. Since the 19th century, trichinellosis has been reported across all continents except Antarctica (Pozio, 2021). In Iran and Turkey, the disease is maintained primarily in a sylvatic cycle, with wild boars and golden jackals identified as key reservoir hosts. Human cases in these regions have been attributed to the consumption of raw or undercooked wild boar meat or contaminated pork products (Borhani et al., 2023). In China, human trichinellosis is predominantly reported in the southwestern part of the country. Between 2009 and 2020, a total of eight outbreaks were recorded, involving 479 cases and two fatalities (Zhang et al., 2022). Notably, as biosecurity measures in commercial pig production have strengthened in many countries, wildlife has become an increasingly important source of human trichinellosis (Malone et al., 2024). Similarly, all 119 cases of trichinellosis documented in Japan between 1974 and 2019 were associated with the consumption of wild bear meat (Murakami et al., 2023).

Benzimidazole derivatives, notably albendazole (ABZ) and mebendazole (MBZ), represent the cornerstone of anthelmintic therapy against trichinellosis and are widely regarded as the most efficacious broad-spectrum agents currently available for this indication (Hanafy et al., 2025). However, the clinical utility of these drugs is constrained by several limitations, including inadequate larvicidal efficacy against the muscle larvae of *Trichinella* spp. and inherently low oral bioavailability. Moreover, despite initial reports of nematode resistance emerging as early as the 1950s (Drudge et al., 1957), the challenge of benzimidazole resistance remains unresolved to date. Therefore, there is a pressing need to develop novel therapeutic agents with enhanced efficacy against trichinellosis.

Against this backdrop, plant-derived extracts from traditional Chinese medicine (TCM) and other ethnobotanical sources have emerged as promising candidates for developing novel anti-*T. spiralis* agents. Their complex phytochemical composition is thought to underlie diverse mechanisms of action, positioning them as viable alternatives or complements to conventional anthelmintic regimens. Consistent with this notion, experimental evidence has demonstrated the efficacy of several botanical extracts against *T. spiralis*. Extracts of *Artemisia vulgaris* and *A*. *absinthium* significantly reduced worm burdens in experimentally infected rats (Caner et al., 2008). *Myrrh* and *thyme* were shown to be effective against both enteral and parenteral phases of *T. spiralis* infection in mice, accompanied by modulation of inducible nitric oxide expression (Attia et al., 2015). In addition, leaf extracts of *Lasia spinosa* significantly decreased adult worm and larval burdens in infected mice, indicating broad-spectrum activity across multiple life-cycle stages (Yadav and Temjenmongla, 2012). These studies support the concept that botanical extracts can act on different developmental stages of *T. spiralis* and modulate host responses, although their precise molecular targets and mechanisms of action remain incompletely understood.

The persistent global burden of trichinellosis, together with the limited efficacy of current benzimidazole-based therapeutics against muscle larvae, underscores the need for novel treatment strategies. Plant-derived natural products with documented antiparasitic activity represent a promising alternative source of anthelmintic candidates, and some have been shown to influence both parasite survival and host tissue responses. However, plant-derived preparations with confirmed efficacy against *Trichinella spiralis* muscle larvae, together with preliminary phytochemical characterization and mechanistic evidence linking parasite injury to host tissue protection, remain insufficiently characterized. The medicinal plants investigated in this study were selected based on traditional use records and reported or presumed antiparasitic relevance. Among them, *Anaphalis margaritacea* ethanol extract (AMEE) showed the most pronounced activity in the preliminary screen and was therefore selected for further investigation. We systematically evaluated its anti-*T. spiralis* activity in vitro and *in vivo*, performed UPLC-Q-TOF-MS/MS-based profiling to obtain preliminary information on its major constituents, and explored its potential mechanism of action through integrated analyses of host tissue responses, quantitative proteomics, mitochondrial enzyme activity assays, and transmission electron microscopy.

## 2 Materials and methods

### 2.1 Preparation of plant extracts and *T. spiralis*

Thirty medicinal plant materials were purchased from Deyin TCM Comprehensive Supermarket (Chengdu, China) and identified by Associate Professor X. L. Miao (Lanzhou Institute of Husbandry and Pharmaceutical Sciences, Chinese Academy of Agricultural Sciences, LIHPS, China). Voucher specimens were deposited in the institutional herbarium. These plants were selected based on traditional medicinal use and reported or putative antiparasitic activity. Two separate 50 g aliquots of each plant material were extracted with purified water and 80% ethanol to obtain the corresponding aqueous extracts and 80% ethanol extracts, respectively. The extracts were concentrated and lyophilized to obtain dry powders. Detailed extraction yields for each plant species are summarized in Supplementary Table S1.

The *T. spiralis* strain was kindly provided by Professor Baoquan Fu from the Lanzhou Veterinary Research Institute, Chinese Academy of Agricultural Sciences (LVRI, China).

### 2.2 Assessment of *T. spiralis* muscle larvae survival in vitro

Kunming mice obtained from Lanzhou Veterinary Research Institute, Chinese Academy of Agricultural Sciences (LVRI, China) were orally inoculated with approximately 600 *T. spiralis* muscle larvae. At 35 days post-infection, mice were euthanized, skeletal muscles were collected, and larvae were recovered by artificial digestion in gastric juice containing 1% pepsin from porcine gastric with an activity of 250 U/mg and 1% HCl at 40 °C for 3 h, followed by sedimentation and washing three times with sterile saline.

The aqueous and 80% ethanol extracts of the 30 medicinal plants were subjected to preliminary in vitro screening at a final concentration of 500 μg/mL to evaluate their larvicidal activity against *T. spiralis* muscle larvae. To ensure a uniform solvent system across the initial screening of all 30 plant extracts, each extract was first prepared as a 10 mg/mL stock solution in DMSO and then diluted to the desired concentration with RPMI-1640 medium supplemented with 10% heat-inactivated fetal bovine serum and 1% penicillin–streptomycin solution, corresponding to 100 U/mL penicillin and 100 μg/mL streptomycin. The final DMSO concentration was kept constant across all groups and did not exceed 1%. In 48-well plates, 50 viable muscle larvae were added to each well, and the total volume was adjusted to 1 mL. Wells containing culture medium with the same final concentration of DMSO served as vehicle controls. The plates were incubated at 37 °C in a humidified atmosphere of 5% CO_2_. Larval viability was assessed at 24, 48, and 72 h after treatment. Larvae were considered dead when they showed no spontaneous movement during a continuous 30 s observation period, no response to gentle mechanical stimulation, and marked morphological changes, including rigidity and loss of transparency. Each treatment was tested in triplicate, and the experiment was independently repeated three times. Extracts showing promising activity in the primary screen were selected for subsequent concentration-response assays based on mortality at the different observation time points.

The mortality of muscle larvae was calculated as follows:

Mortality(%)=(numberofdeadlarvae/totalnumberoflarvae)×100

### 2.3 UPLC-Q-TOF-MS/MS analysis of AMEE

AMEE was analyzed by UPLC-Q-TOF-MS/MS for preliminary chemical characterization. Briefly, 5 mg of AMEE dry powder was dissolved in methanol in a 10 mL volumetric flask, sonicated, centrifuged, and filtered through a 0.22 μm membrane before analysis. Chromatographic separation was performed on a Luna® Omega 1.6 μm Polar C18 column (100 mm × 2.1 mm, Phenomenex) at 0.40 mL/min with a total run time of 20 min. The mobile phase consisted of 0.1% formic acid in water (A) and acetonitrile (B), using the following gradient: 0-2.0 min, 70% A; 2.0-15.0 min, linear gradient to 5% A; 15.0-17.0 min, 5% A; and 17.1-20.0 min, returned to 70% A for re-equilibration. Mass spectrometric data were acquired using a DuoSpray electrospray ionization source in negative ion mode. TOF-MS and IDA-TOF-MS/MS were used to obtain precursor and fragment ion information for compound annotation. The ion spray voltage was set at −4500 V, the source temperature at 500 °C, the declustering potential at −80 V, and the collision energy at −40 eV with a collision energy spread of ±20 eV. The TOF-MS and TOF-MS/MS scan ranges were m/z 100-1000 and 50-1000, respectively.

### 2.4 Therapeutic effects of AMEE on *T. spiralis* infection in mice

**Fig. 1:**
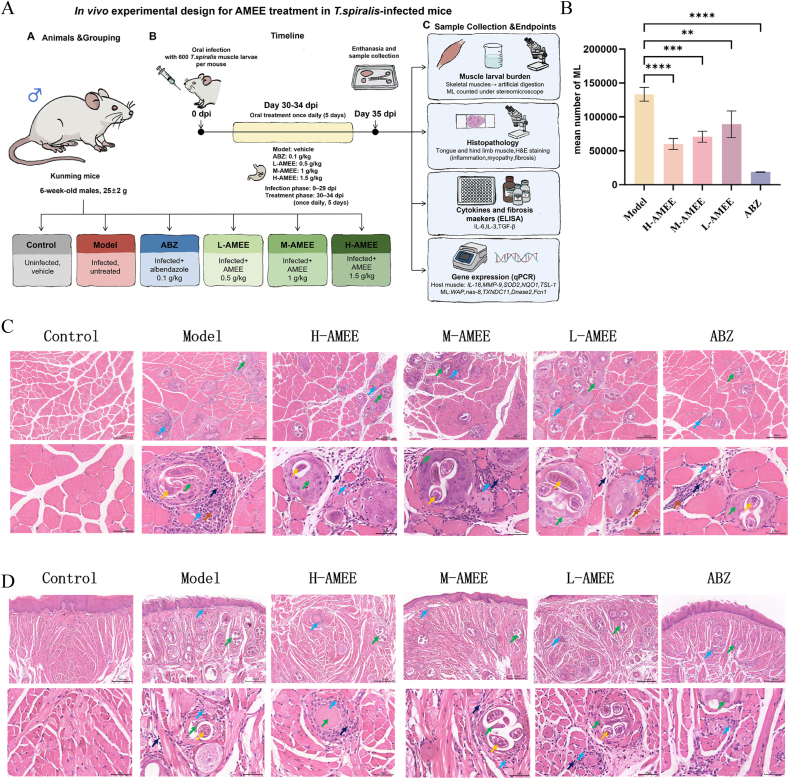
Effects of AMEE on parasite burden and muscle histopathology in *T. spiralis*-infected mice. (A) Schematic diagram of experimental design.(B) Muscle larvae burden in different treatment groups (control, model, ABZ, L-AMEE, M-AMEE and H-AMEE). Data are expressed as mean ± SD (n = 10). P < 0.05 vs infected model group. (C) H&E-stained sections of leg muscle showing inflammatory cell infiltration and larval capsules. (D) H&E-stained sections of tongue muscle. Scale bars = 50 μm and 200 μm.

Based on the in vitro screening results, AMEE was selected for *in vivo* evaluation. A total of 60 six-week-old male Kunming mice (weighing 25 ± 2 g) were orally inoculated with approximately 600 muscle larvae of *T. spiralis*. Male mice were used to reduce potential variability related to sex differences. The mice were randomly assigned to six groups (n = 10 per group): H-AMEE (1.5 g/kg), M-AMEE (1.0 g/kg), L-AMEE (0.5 g/kg), ABZ (0.1 g/kg), model (infected, untreated), and control (uninfected). The AMEE doses were selected based on preliminary *in vivo* observations and to allow evaluation of dose-dependent effects. ABZ was used as a positive control because of its established anti-*Trichinella* activity. Treatment was initiated at 30 days post-infection (dpi), when muscle larvae were fully established in skeletal muscle, to target the muscle stage specifically, and was continued for 5 consecutive days until necropsy at 35 dpi (Fig. 1A). Subsequently, muscle samples from each group were individually digested using artificial gastric juice. The resulting larvae were enumerated under a stereomicroscope at 40× magnification, and larval burden was expressed as the total number of larvae recovered per mouse.

### 2.5 Histopathology

Tissue samples from the tongue and hind limb muscles of mice were fixed in 4% paraformaldehyde, embedded in paraffin, and sectioned. The sections were then stained with hematoxylin and eosin (H&E), and histological alterations, including the severity of inflammatory cell infiltration, myofiber degeneration and necrosis, and pericystic tissue remodelling, were examined under a light microscope to evaluate the extent of tissue injury.

### 2.6 Determination of cytokine levels in muscle tissue

Muscle tissue samples were collected from each mouse, weighed, and homogenized on ice in PBS at a ratio of 1:9 (w/v). The homogenates were centrifuged at 12,000 × g for 10 min at 4 °C, and the supernatants were collected for cytokine determination. The levels of interleukin-6 (IL-6), IL-13, and TGF-β in muscle tissue homogenates were measured using commercial ELISA kits from Shanghai Jianglai Biotechnology Co., Ltd. according to the manufacturer's instructions. Briefly, standards and tissue homogenate supernatants were added to the ELISA plates, incubated with the corresponding enzyme conjugates and chromogenic substrates, and the absorbance was measured at 450 nm using a microplate reader (Thermo Fisher Scientific, USA). Each sample was assayed in duplicate, and cytokine levels were calculated from standard curves.

### 2.7 Analysis by quantitative real-time PCR (qPCR)

Total RNA was isolated from muscle tissue using a commercial RNA extraction kit (Sparkjade, China) and subsequently reverse-transcribed into complementary DNA (cDNA) following the manufacturer's protocol of a reverse transcription PCR kit (Sparkjade, China). Gene-specific primers were designed and synthesized for quantitative PCR analysis. The primer sequences are listed in Table S4.

Gene expression levels were quantified using the 2^−ΔΔCT^ method. All qPCR reactions were performed in triplicate to ensure technical reproducibility.

### 2.8 Proteomic analysis and verification

*T. spiralis* muscle larvae were cultured in RPMI-1640 medium supplemented with 10% heat-inactivated fetal bovine serum and 1% penicillin–streptomycin in the presence of AMEE (500 μg/mL) for 3 days at 37 °C in a humidified atmosphere of 5% CO_2_. AMEE was dissolved in water before addition to the culture system. Control larvae were cultured in the same medium under identical conditions but without AMEE. After incubation, larvae were collected and washed repeatedly before proteomic analysis to reduce residual non-parasite material, and were then stored at −80 °C until analysis. Proteomic analysis was performed using TMT-based quantitative proteomics. Proteins were extracted from muscle larvae, digested using the filter-aided sample preparation (FASP) method, labeled with TMT reagents, fractionated by high-pH reverse-phase liquid chromatography, and analyzed by LC-MS/MS. DDA data were processed using MaxQuant, and DIA data were analyzed using DIA-NN, with a false discovery rate (FDR) threshold of 1%. Functional annotation of the identified proteins was performed using Gene Ontology (GO), KEGG, Pfam, and subcellular localization databases. Differentially expressed proteins were subjected to functional enrichment analysis using Fisher's exact test (P < 0.05). Hierarchical clustering and protein–protein interaction network analysis were subsequently performed, with the STRING database used for PPI analysis. Proteomic analysis was conducted using three independent biological replicates per group.

To verify the proteomic findings at the transcriptional level, quantitative real-time PCR (qPCR) was performed on selected genes predicted to be differentially expressed based on proteomic screening. These genes were selected according to the magnitude of differential expression and their potential relevance to parasite stress responses, secretion-related functions, or mitochondrial-associated processes. The primer sequences are listed in Table S4. Gene expression levels were calculated using the 2^^−ΔΔCT^ method. All qPCR reactions were performed in triplicate.

### 2.9 Determination of mitochondrial enzyme activity

*T. spiralis* muscle larvae were recovered from infected mice by artificial digestion, washed three times with sterile saline, and counted. Viable muscle larvae were then suspended in RPMI-1640 medium supplemented with 10% heat-inactivated fetal bovine serum and 1% penicillin–streptomycin, and approximately 5000 larvae were added to each well. AMEE was first dissolved in water and then serially diluted with culture medium to final concentrations of 500, 100, and 50 μg/mL, designated as the high-concentration (H-AMEE), medium-concentration (M-AMEE), and low-concentration (L-AMEE) groups, respectively. These concentrations were selected based on preliminary in vitro concentration-response experiments, in which AMEE at 100 and 500 μg/mL caused complete muscle larvae mortality within 72 h, whereas 50 μg/mL produced partial larvicidal activity. Albendazole (ABZ, 100 μg/mL) was included as a positive control based on preliminary in vitro optimization and solubility considerations, and culture medium alone served as the untreated control. All groups were incubated at 37 °C in a humidified atmosphere of 5% CO_2_ for 3 days, after which larvae were collected for subsequent analyses. Larvae were homogenized, and the activities of mitochondrial respiratory chain complexes were determined in whole-larval homogenates and normalized to total protein concentration. The activities of complex I (NADH:ubiquinone oxidoreductase), complex II (succinate dehydrogenase), complex III (ubiquinol-cytochrome c reductase), complex IV (cytochrome *c* oxidase), and complex V (ATP synthase) were measured using enzyme activity assay kits (Beijing Saierbo Technology Co., Ltd., Beijing, China).

### 2.10 Transmission electron microscopy examination

After 72 h of in vitro treatment, muscle larvae from the H-AMEE-treated group and the untreated control group were collected and processed for transmission electron microscopy (TEM). Samples were fixed in glutaraldehyde, post-fixed in osmium tetroxide, dehydrated through a graded acetone series, and embedded in epoxy resin. Ultrathin sections were prepared and double-stained with uranyl acetate and lead citrate before TEM observation.

### 2.11 Statistical analysis

Statistical analyses for larval burden, ELISA measurements, qPCR data, and mitochondrial enzyme activity assays were performed using GraphPad Prism version 10.1.2. Data are presented as mean ± standard deviation (SD). Multiple-group comparisons were performed using one-way analysis of variance (ANOVA) followed by Tukey's multiple-comparisons test. A value of p < 0.05 was considered statistically significant. For proteomic analysis, differentially expressed proteins were screened using a fold-change threshold of >1.5 together with an FDR-adjusted p value < 0.05. Functional enrichment analysis of differentially expressed proteins was performed using Fisher's exact test, with p < 0.05 considered significant. Histopathological and TEM observations were analyzed descriptively.

## 3 Results

### 3.1 In vitro larvicidal activity of plant extracts against muscle larvae

Initial screening at 500 μg/mL identified eleven plant extracts with in vitro larvicidal activity against *T. spiralis* muscle larvae, including eight 80% ethanol extracts from *Anaphalis margaritacea*, *Gelsemium elegans*, *Leontopodium leontopodioides*, *Melia azedarach*, *Mirabilis jalapa*, *Phyllanthus emblica*, *Plumbago zeylanica*, and *Sargentodoxa cuneata*, as well as three aqueous extracts from *Punica granatum*, *Juglans regia*, and *Taxus wallichiana* (Table S2). Among these active extracts, AMEE showed the most rapid larvicidal activity and remained active at lower concentrations (Table 1). AMEE was therefore selected for subsequent chemical characterization, *in vivo* evaluation, and mechanistic investigation.

**Table 1:** Concentration–response of selected plant extracts against *T. spiralis* muscle larvae in vitro*.*

	Concentration (μg/mL)	Larvicidal activity (%)		
		24h	48h	72h
*Anaphalis margaritacea* 80%EE	500	85.82 ± 5.35	95.06 ± 7.24	100 ± 0.00
	100	33.11 ± 4.79	81.04 ± 5.80	100 ± 0.00
	50	17.46 ± 5.38	58.35 ± 4.58	85.38 ± 5.52
*Carpesium abrotanoides* 80%EE	500	14.28 ± 1.02	65.65 ± 2.11	82.03 ± 6.90
	100	-	-	-
	50	-	-	-
*Gelsemium elegans* 80%EE	500	2.82 ± 0.79	46.03 ± 5.21	71.23 ± 8.52
	100	-	-	-
	50	-	-	-
*Leontopodium leontopodioides* 80%EE	500	9.15 ± 3.20	69.87 ± 7.67	91.06 ± 7.92
	100	8.79 ± 4.71	46.53 ± 3.15	50.99 ± 4.52
	50	-	-	-
*Melia azedarach* 80%EE	500	50.73 ± 6.78	84.72 ± 9.15	100 ± 0.00
	100	-	-	-
	50	-	-	-
*Mirabilis jalapa* 80%EE	500	-	26.09 ± 7.96	78.69 ± 4.37
	100	-	-	-
	50	-	-	-
*Phyllanthus emblica* 80%EE	500	35.28 ± 6.72	97.32 ± 9.25	100 ± 0.00
	100	12.95 ± 4.72	30.64 ± 7.53	38.99 ± 7.23
	50	-	-	-
*Plumbago zeylanica* 80%EE	500	7.2 ± 2.25	75.17 ± 8.42	100 ± 0.00
	100	-	-	66.91 ± 5.69
	50	-	-	-
*Punica granatum* AE	500	61.81 ± 6.03	93.09 ± 6.82	96.07 ± 4.79
	100	17.38 ± 2.89	62.19 ± 7.67	78.70 ± 6.01
	50	-	-	-
*Sargentodoxa cuneata* 80%EE	500	5.76 ± 1.04	71.22 ± 6.52	91.06 ± 7.92
	100	8.07 ± 3.54	30.34 ± 4.20	36.65 ± 7.30
	50	-	24.28 ± 3.05	35.44 ± 6.40
*Semen Juglandis* AE	500	11.49 ± 1.69	62.05 ± 5.22	68.74 ± 4.82
	100	-	21.15 ± 4.33	41.32 ± 8.57
	50	-	-	-
*Taxus wallichiana var. chinensis* AE	500	4.78 ± 2.76	44.71 ± 2.64	73.42 ± 6.28
	100	-	33.54 ± 4.30	38.26 ± 5.51
	50	-	-	-

Data are expressed as larval mortality (%) at 24, 48 and 72 h “–”, no detectable.

### 3.2 UPLC-Q-TOF-MS/MS profiling of AMEE

UPLC-Q-TOF-MS/MS analysis was performed to characterize the major constituents of AMEE. Representative total ion chromatograms are shown in Fig. S1 and S2. Based on accurate mass measurement and MS/MS library matching in the negative ion mode, eight major constituents were tentatively identified (Table 2). These compounds mainly included phenolic acids, other organic acids, and flavonoids, namely protocatechuic aldehyde (m/z 137.0240), quinic acid (m/z 191.0560), rosmarinic acid (m/z 359.0770), eriodictyol (m/z 287.0560), luteolin (m/z 285.0404), apigenin (m/z 269.0450), baicalin (m/z 445.0780), and genistin (m/z 431.0980). The small mass errors and high library matching scores supported the reliability of these tentative assignments. Overall, these results indicate that organic acids and flavonoids are major constituents of AMEE.

**Table 2:** Major compounds tentatively identified by UPLC-Q-TOF-MS/MS.

No.	Retention time (min)	Ion species	Molecular formula	MS	MS/MS	Error (ppm)	Library score	Compound name
1	1.23	[M-H]-	C_21_H_20_O_10_	431.098	270.0456; 269.0444; 268.0373; 432.0947	−0.8	99.4	Genistin
2	0.98	[M-H]-	C_7_H_6_O_3_	137.024	136.0170; 108.0221	1.5	99	Protocatechuic aldehyde
3	2.57	[M-H]-	C_15_H_12_O_6_	287.056	151.0033; 135.0447; 136.0455	0.1	98.9	Eriodictyol
4	0.78	[M-H]-	C_18_H_16_O_8_	359.077	179.0342; 161.0245; 197.0467; 135.0460	−2.3	98.6	Rosmarinic acid
5	0.49	[M-H]-	C_7_H_12_O_6_	191.056	173.0459; 127.0405	−0.7	97.8	Quinic acid
6	2.9	[M-H]-	C_15_H_1_0O_6_	285.04	201.0154; 151.0018; 133.0293	−0.2	97.7	Luteolin
7	0.77	[M-H]-	C_21_H_18_O_11_	445.078	269.0446; 270.0442; 246.0347; 175.0263	−1.7	97.1	Baicalin
8	4.29	[M-H]-	C_15_H_10_O_5_	269.045	151.0040; 117.0358; 225.0519; 255.8389	−0.2	95	Apigenin

Note: Ion species represent the adduct forms detected in negative electrospray ionization (ESI) mode. MS denotes the accurate mass of the precursor ion, and MS/MS denotes the major fragment ions. Error is expressed as mass error (ppm). Library score indicates the matching score between the sample MS/MS spectrum and the library spectrum.

### 3.3 AMEE reduces muscle larvae burden *in vivo*

The administration of AMEE resulted in a dose-dependent reduction in muscle larvae burden. Compared with the untreated infected group, the H-AMEE, M-AMEE, and L-AMEE groups exhibited larval reductions of 59.67%, 44.77%, and 26.78%, respectively, with mean larval burdens of 60,120, 70,667, and 89,086 larvae per mouse, respectively (Fig. 1B). In contrast, the ABZ-treated group showed a more pronounced reduction of 84.48%.

### 3.4 AMEE ameliorates histopathological changes in *T. spiralis*-infected mice

As shown in Fig. 1C and D, histopathological examination of tongue and hind limb muscles revealed differential pathological manifestations across experimental groups. Control groups displayed preserved muscular architecture with well-organized fibers and no signs of degeneration or inflammatory infiltration. In contrast, the model and ABZ groups exhibited prominent parasitic encapsulation, characterized by fibrous-walled cysts containing necrotic muscle fibers within a lightly stained matrix. These regions showed cellular alterations including nuclear enlargement and distinct nucleoli, accompanied by inflammatory infiltrates composed predominantly of neutrophils, lymphocytes, and monocytes. Pericystic areas demonstrated myofibrillar dissolution and structural disintegration. Conversely, all AMEE-treated groups (H-AMEE, M-AMEE, L-AMEE) showed markedly attenuated pathology, with only focal tissue necrosis and minimal inflammatory responses around scattered cysts, indicating a dose-dependent protective effect of AMEE against *T. spiralis*-induced myopathy.

### 3.5 AMEE attenuates the inflammatory response in *T. spiralis*-infected mice

**Fig. 2:**
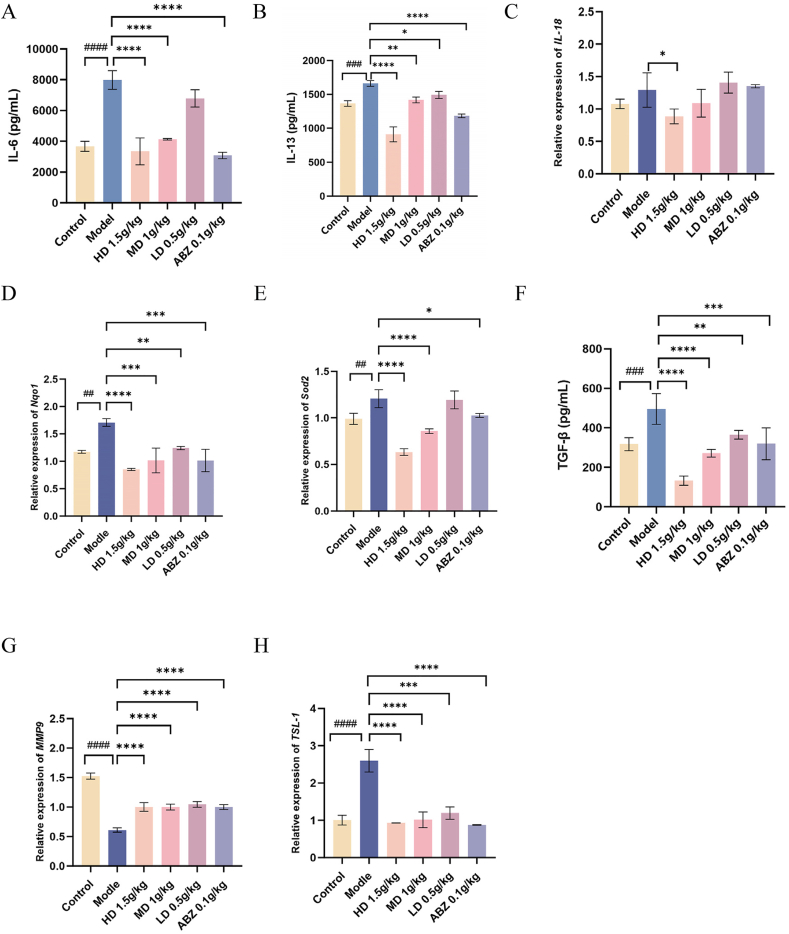
Effects of AMEE on cytokine and oxidative stress-related gene expression in skeletal muscles of *T. spiralis*-infected mice. (A) IL-6, (B) IL-13 and (C) *IL-18* levels. (D) *Nqo1* and (E) *Sod2* mRNA expression. (F) TGF-β, (G) *MMP-9* and (H) *TSL-1* levels. Data are expressed as mean ± SD (n = 10). ∗P < 0.05, ∗∗P < 0.01, ∗∗∗P < 0.001, ∗∗∗∗P < 0.0001 vs infected model group.

The levels of IL-6 (Fig. 2A) and IL-13 (Fig. 2B) were significantly increased in the infected model group compared with the control group. AMEE treatment significantly reduced the levels of both cytokines, and this effect was more pronounced in the medium- and high-dose groups. In particular, IL-6 levels was markedly reduced in the H-AMEE and M-AMEE groups relative to the model group, whereas the reduction in the L-AMEE group was less pronounced. IL-13 showed a similar pattern, with the H-AMEE group exhibiting the greatest decrease among the AMEE-treated groups. In addition, *IL-18* expression (Fig. 2C) was significantly reduced only in the H-AMEE group, while the decrease in the M-AMEE group did not reach statistical significance. These results indicate that AMEE alleviated the inflammatory response induced by *T. spiralis* infection in skeletal muscle.

### 3.6 AMEE attenuates oxidative stress in *T. spiralis*-infected mice

The mRNA expression levels of *Nqo1* (Fig. 2D) and Sod2 (Fig. 2E) were significantly elevated in the skeletal muscle of infected mice compared with those in the control group. AMEE treatment reduced the expression of both oxidative stress-related genes, although the magnitude of the effect differed between them. For *Nqo1*, significant downregulation was observed in the medium- and high-dose groups, with the H-AMEE group showing the strongest reduction relative to the model group. In contrast, *Sod2* expression showed a weaker response, and significant downregulation was observed only in the H-AMEE group. ABZ treatment also reduced the expression of these two genes to varying degrees. These findings suggest that AMEE partially reversed the oxidative stress-associated transcriptional changes induced by *T. spiralis* infection.

### 3.7 AMEE attenuates fibrosis in *T. spiralis*-infected mice

Compared with the control group, the infected model group showed significantly increased expression of TGF-β (Fig. 2F) and significantly decreased expression of *MMP-9* (Fig. 2G). AMEE treatment significantly reduced TGF-β expression and increased *MMP-9* expression, with the high-dose group showing the most pronounced effect. Relative to the model group, TGF-β expression was reduced most markedly in the H-AMEE group, whereas the medium- and low-dose groups showed weaker but still significant effects. For *MMP-9*, all AMEE-treated groups and the ABZ group showed increased expression compared with the model group, indicating partial reversal of the infection-associated fibrosis-related changes. In parallel, *TSL-1* expression (Fig. 2H) was decreased after AMEE treatment, consistent with the reduced larval burden observed in infected mice. These results indicate that AMEE treatment was associated with attenuation of fibrosis-related pathological changes in skeletal muscle during *T. spiralis* infection.

### 3.8 Proteomics analysis

**Fig. 3:**
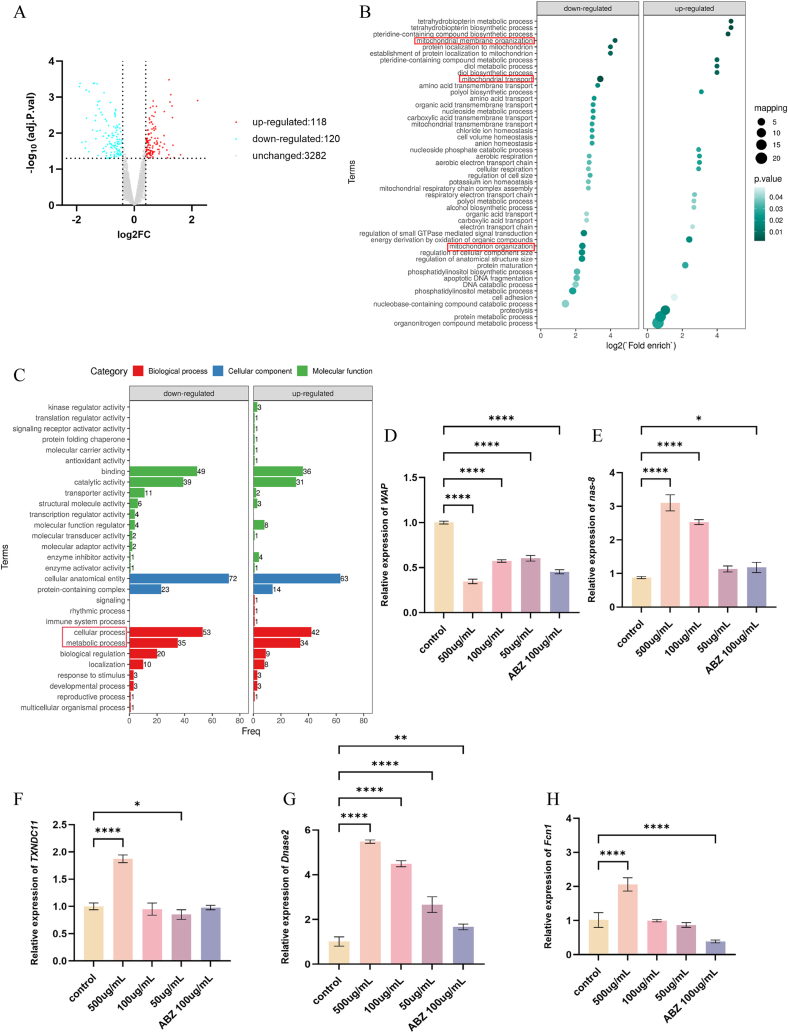
Proteomic profiling and transcriptional validation of AMEE-treated *T. spiralis* muscle larvae. (A) Volcano plot of differentially expressed proteins, (B) Gene Ontology (GO) classification of differentially expressed proteins. (C) GO biological process enrichment analysis. Relative mRNA expression levels of (D) *WAP*, (E) *nas-8*, (F) *TXNDC11*, (G) *Dnase2* and (H) *Fcn1* in muscle larvae as determined by qPCR.

Proteomic analysis identified a total of 3592 proteins in *T. spiralis* muscle larvae. Among these, 238 proteins were differentially expressed in the AMEE-treated group compared with the untreated control group, based on the screening criteria of fold change >1.5 and FDR <0.05, including 118 upregulated proteins and 120 downregulated proteins. Functional enrichment analysis showed that several biological processes were significantly altered after AMEE treatment. As shown in Fig. 3A, the downregulated proteins were mainly enriched in categories related to cellular and metabolic processes. Heatmap analysis further showed that multiple mitochondrial-associated processes, including mitochondrial membrane organization and mitochondrial transport, were downregulated after AMEE treatment (Fig. 3B). These results indicate that AMEE treatment was associated with marked alterations in mitochondrial-related and metabolic pathways in *T. spiralis* muscle larvae.

### 3.9 Validation of proteomics-identified candidate genes in *T. spiralis* muscle larvae

To verify the expression changes of selected candidates identified in the proteomic analysis (Table S3), the mRNA levels of several genes in *T. spiralis* muscle larvae were examined by qPCR (Table S4). AMEE treatment significantly reduced the expression of *WAP* (Fig. 3D). In the H-AMEE group, *nas-8*, *TXNDC11*, and *Dnase2* were significantly upregulated (Fig. 3E–G), with *Dnase2* showing the greatest increase and rising by more than fourfold relative to the control group. In addition, opposite expression patterns of *Fcn1* were observed following AMEE and ABZ treatment, with *Fcn1* mRNA being increased in the H-AMEE group but significantly decreased in the ABZ group (Fig. 3H). These results were generally consistent with the proteomic findings and further supported that AMEE affected multiple biological processes in *T. spiralis* muscle larvae.

### 3.10 Effect of AMEE on mitochondrial respiratory chain complexes of *T*. *spiralis*

**Fig. 4:**
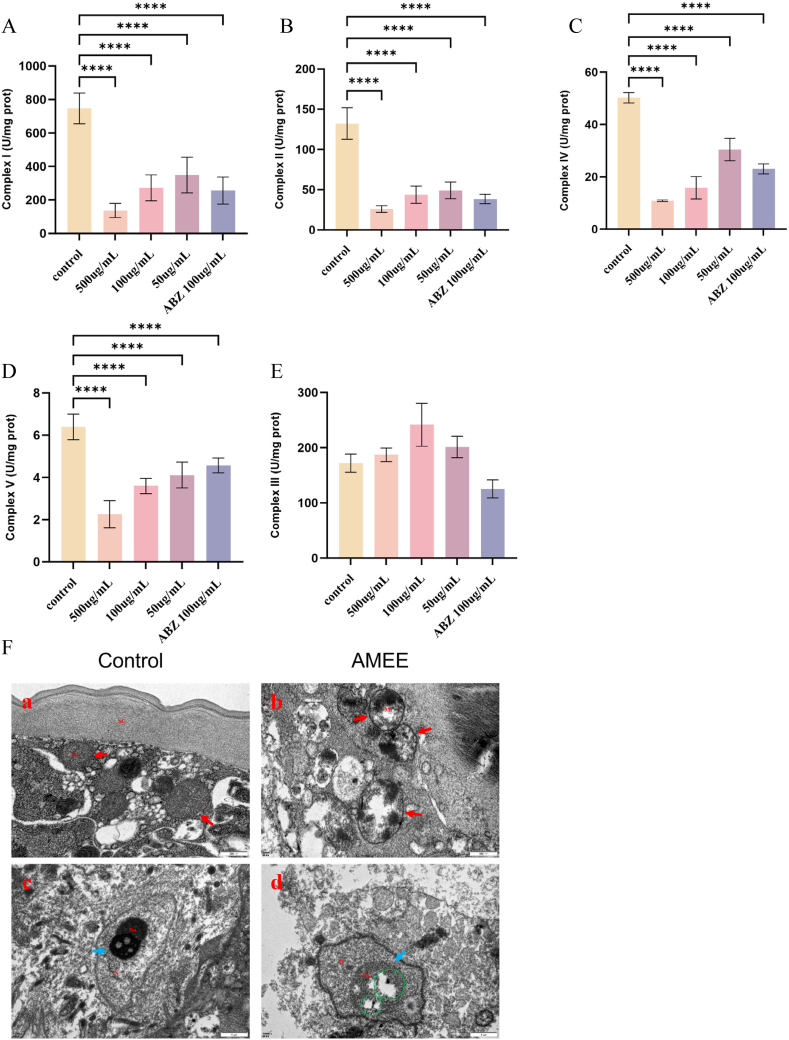
Activities of mitochondrial respiratory chain complexes in *T. spiralis* muscle larvae after AMEE treatment. (A) Complex I, (B) Complex II, (C) Complex IV, (D) Complex V, and (E) Complex III. Data are expressed as mean ± SD. ∗P < 0.05, ∗∗P < 0.01, ∗∗∗P < 0.001, ∗∗∗∗P < 0.0001 vs control group. Transmission electron micrographs of *T. spiralis* muscle larvae are shown in Fa–Fd. Transmission electron micrographs of muscle larvae. (Fa) Normal morphology showing stratum corneum (SC) and intact mitochondria (Mi), ×30,000. (Fb) Nucleus (N) and nucleolus (No) with intact nuclear membrane and relatively electron-dense nucleolus, ×15,000. (Fc) Mitochondrial swelling (Mi, arrows) after AMEE treatment, ×30,000. (Fd) Nucleus (N) and nucleolus (No) showing reduced nucleolar electron density and areas of component degradation (circles), ×15,000.

The activities of mitochondrial respiratory chain complexes were measured to further assess the effect of AMEE on mitochondrial function in *T. spiralis* muscle larvae. Compared with the untreated control group, AMEE treatment significantly reduced the activities of complex I (Fig. 4A), complex II (Fig. 4B), and complex IV (Fig. 4C). In the AMEE-treated groups, the activities of complexes I, II, IV, and V were significantly reduced, with the strongest inhibitory effect observed in the H-AMEE group. In contrast, no consistent or significant change was observed in complex III activity (Fig. 4E). Among the AMEE-treated groups, the high-dose group showed the most pronounced inhibitory effect on mitochondrial respiratory chain enzyme activities. These results indicate that AMEE markedly affected multiple components of the mitochondrial respiratory chain in *T. spiralis* muscle larvae.

### 3.11 Effect of AMEE on mitochondrial ultrastructure of *T. spiralis* muscle larvae

Transmission electron microscopy showed marked ultrastructural differences between the untreated control group and the AMEE-treated group. In the control group, *T. spiralis* muscle larvae maintained an intact ultrastructure, with a continuous cuticle and cell membrane, well-preserved mitochondria with clearly visible cristae, and nuclei with regular morphology and intact nuclear envelopes (Fig. 4Fa,b). In contrast, larvae in the AMEE-treated group showed obvious subcellular alterations despite retaining an outwardly intact cuticle (Fig. 4Fc,d). Portions of the cell membrane appeared disrupted, and mitochondria exhibited pronounced swelling, cristae fragmentation or loss, and matrix rarefaction or vacuolization. In addition, nuclear morphology became irregular, and the nucleolus showed structural disruption with localized electron-lucent areas. These observations indicate that AMEE induced marked mitochondrial and subcellular damage in *T. spiralis* muscle larvae.

## 4 Discussion

Trichinellosis remains an important food-borne zoonosis worldwide, with wildlife-associated transmission receiving increasing attention in recent years (Borhani et al., 2023; Zhang et al., 2022; Malone et al., 2024; Murakami et al., 2023). The muscle-larval stage remains difficult to treat because larvae are embedded in skeletal muscle and are less accessible to conventional anthelmintic therapy (Bilska-Zając et al., 2021; Pozio, 2021). Benzimidazole drugs, including albendazole and mebendazole, remain the mainstay of treatment, but their efficacy against established muscle larvae is limited. Their pharmacological action is mainly related to β-tubulin binding and disruption of microtubule-dependent processes, which differs from the mitochondrial changes observed after AMEE treatment in the present study (Lacey, 1988; Lubega and Prichard, 1990; Hanafy et al., 2025). Therefore, the identification of new plant-derived candidates with activity against *T. spiralis* muscle larvae remains necessary.

AMEE showed clear anti-*T. spiralis* activity both in vitro and *in vivo*. As the most active extract identified from the initial screen of 30 medicinal plant extracts, AMEE caused complete larval mortality within 72 h at 100 μg/mL, which supported its selection for further mechanistic investigation. In the murine infection model, the high-dose AMEE group reduced muscle larvae burden by 59.67%, although this effect remained lower than that of ABZ. Notably, AMEE also alleviated histopathological injury in infected muscle, as reflected by reduced inflammatory infiltration and attenuation of tissue damage around larval cysts. Taken together, these findings suggest that AMEE treatment was associated not only with reduced parasite burden but also with alleviation of infection-associated muscle pathology.

Preliminary UPLC-Q-TOF-MS/MS analysis indicated that AMEE contains multiple phenolic acids, other organic acids, and flavonoids, including rosmarinic acid, protocatechuic aldehyde, quinic acid, eriodictyol, luteolin, apigenin, baicalin, and genistin. Previous phytochemical studies have also reported flavonoids, terpenoids, and related secondary metabolites in *A. margaritacea* (Wollenweber et al., 1993; Ahmed et al., 2004; Ren et al., 2009). Given the recognised antiparasitic relevance of plant secondary metabolites, these findings provide a chemical basis for subsequent activity-guided studies (Wink, 2012; ElGhannam et al., 2023). However, the present chemical analysis remains preliminary, and the active constituents responsible for the anti-*T. spiralis* activity of AMEE require further bioassay-guided fractionation and structural characterisation.

The host-response results were generally consistent with the histopathological observations. *T. spiralis* infection is known to induce marked host inflammatory and type 2 immune responses, which are closely related to parasite persistence, tissue repair, and fibrosis-related remodelling (Ding et al., 2017; Ashour, 2013; Briggs et al., 2018; Gieseck et al., 2018). In the present study, IL-6 and IL-13 levels were increased in infected muscle, whereas AMEE treatment reduced both cytokines, especially in the higher-dose groups. In addition, AMEE partially reversed the infection-induced changes in TGF-β and *MMP-9*, suggesting an association with reduced fibrosis-related responses in infected muscle. TGF-β and IL-13 are closely involved in tissue remodelling and fibrotic responses (Gieseck et al., 2018; Roeb, 2023). However, the current data cannot determine whether these host-response changes resulted from direct regulation of host immune pathways or from the decrease in larval burden. Therefore, these results should be interpreted as treatment-associated changes rather than direct evidence of host-targeted immunomodulation.

Oxidative stress-related markers were also altered after AMEE treatment. In infected mice, *Nqo1* and *Sod2* expression was elevated, indicating activation of antioxidant defence-related transcriptional responses in skeletal muscle. *NQO1* is an important redox-regulatory enzyme, whereas *Sod2* is involved in mitochondrial superoxide detoxification (Ross and Siegel, 2021; Zorova et al., 2018). AMEE treatment reduced the expression of these markers, especially in the high-dose group, suggesting that AMEE treatment was associated with attenuation of oxidative stress-related responses in infected muscle. However, because only transcriptional markers were measured, direct oxidative stress indicators such as ROS levels, lipid peroxidation products, or antioxidant enzyme activities would be needed to further confirm this effect.

The integrated proteomic, enzymatic, and ultrastructural data suggest that mitochondrial dysfunction is involved in the anti-*T. spiralis* activity of AMEE. Proteomic analysis revealed that AMEE treatment altered the expression of multiple proteins involved in cellular and metabolic processes, including mitochondrial-associated pathways. Consistently, AMEE reduced the activities of mitochondrial respiratory chain complexes I, II, IV, and V, whereas complex III showed no consistent or significant change. Because oxidative phosphorylation depends on coordinated electron transfer through the respiratory chain and proton-motive force generation, inhibition of these complexes may compromise mitochondrial energy metabolism (Zorova et al., 2018; Kohler et al., 2023). TEM observations showing mitochondrial swelling, cristae disruption, and vacuolization provided morphological support for mitochondrial injury after AMEE treatment (Bughdadi, 2010; Joubert et al., 2021). Together, these results indicate that AMEE-induced larval injury is associated with mitochondrial dysfunction, although the current evidence does not prove that mitochondria are the direct molecular target of AMEE.

**Fig. 5:**
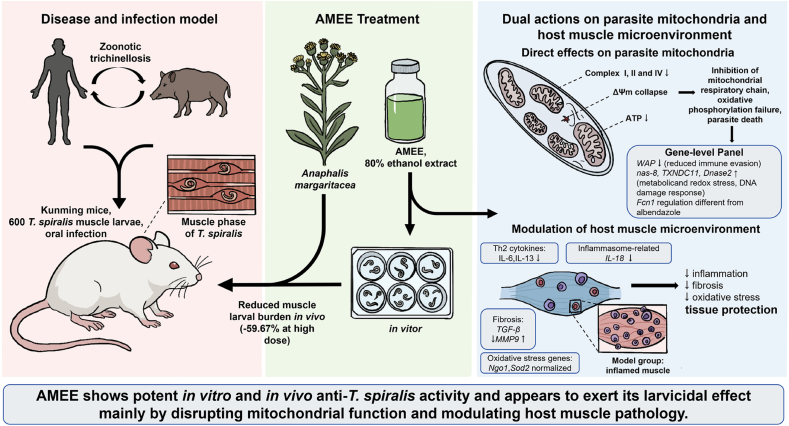
*Anaphalis margaritacea* ethanol extract exhibits potent anti-*Trichinella spiralis* activity via mitochondrial dysfunction and host tissue protection.

Previous studies have reported anti-*T. spiralis* activity for various plant-derived extracts, including *Artemisia vulgaris*, *Artemisia absinthium*, *myrrh, thyme*, and *Lasia spinosa* (Caner et al., 2008; Attia et al., 2015; Yadav and Temjenmongla, 2012). In contrast, the present study not only demonstrated the in vitro and *in vivo* anti-*T. spiralis* activity of AMEE, but also combined preliminary chemical characterization with analysis of host inflammatory, oxidative stress-related, and fibrosis-related changes, together with proteomic profiling, mitochondrial respiratory chain enzyme assays, and ultrastructural observations. These findings provide a broader experimental basis for further investigation of AMEE as a plant-derived candidate against trichinellosis. A schematic summary of the experimental design and the proposed effects of AMEE on *T. spiralis* and host muscle pathology is shown in Fig. 5.

Several limitations of the present study should be considered. The *in vivo* experiments were conducted in a single mouse strain using one laboratory-maintained *T. spiralis* isolate, so the generalizability of the findings remains to be established. AMEE was evaluated as a crude ethanol extract, and the specific constituents responsible for its biological effects remain undefined. Larval viability in vitro was assessed using morphological and motility-based criteria rather than dye-based or metabolic viability assays. In addition, although the combined proteomic, enzymatic, and ultrastructural findings support the involvement of mitochondrial dysfunction in AMEE action, direct measurements of parasite ATP content, mitochondrial membrane potential, and ROS production would further strengthen the mechanistic interpretation.

In conclusion, the present study shows that *A. margaritacea* ethanol extract has significant anti-*T. spiralis* activity in vitro and *in vivo*. The data suggest that AMEE-induced larval injury is associated with mitochondrial dysfunction and that AMEE treatment is accompanied by attenuation of infection-associated muscle pathology.

## CRediT authorship contribution statement

**Xiaoyan Yu:** Writing – original draft, Visualization, Methodology, Investigation, Formal analysis, Data curation, Conceptualization. **Xinyuan Cao:** Writing – review & editing, Investigation, Formal analysis, Data curation. **Yuchao Ma:** Writing – review & editing, Validation, Methodology, Investigation. **Lixia Dai:** Writing – review & editing, Investigation, Formal analysis, Data curation. **Tong Bu:** Resources, Investigation. **Xiaorong Yang:** Resources, Investigation, Data curation. **Zile Gong:** Resources, Investigation. **Jianing Pang:** Investigation, Formal analysis. **Yang Huang:** Investigation, Formal analysis. **Mingze Cao:** Investigation, Formal analysis. **Zhen Zhu:** Writing – review & editing, Supervision, Project administration, Methodology, Conceptualization. **Xiaolou Miao:** Writing – review & editing, Supervision, Project administration, Methodology, Conceptualization. **Xiaofei Shang:** Writing – review & editing, Supervision, Project administration, Methodology, Conceptualization.

## Ethics statement

All experimental procedures involving animals were reviewed and approved by the Animal Ethics Committee of the Lanzhou Institute of Husbandry and Pharmaceutical Sciences, Chinese Academy of Agricultural Sciences (Approval No. 2025-65). The study was conducted in accordance with national and institutional guidelines for the care and use of laboratory animals, and all efforts were made to minimise animal suffering and to reduce the number of animals used.

## Conflict of interest statement

The authors declare that they have no known competing financial interests or personal relationships that could have appeared to influence the work reported in this paper.

## Data Availability

The proteomics dataset generated in this study has been deposited in the figshare repository and is publicly available at https://doi.org/10.6084/m9.figshare.31981338.v1.
