# Supplementary material for: Anaphalis margaritacea ethanol extract exhibits potent anti-Trichinella spiralis activity via mitochondrial dysfunction and host tissue protection
Source: Int J Parasitol Drugs Drug Resist. 2026 May 18;31:100650. doi: 10.1016/j.ijpddr.2026.100650 (PMC13253213; doi:10.1016/j.ijpddr.2026.100650)
Supplement: Multimedia component 1 [file mmc1.docx]

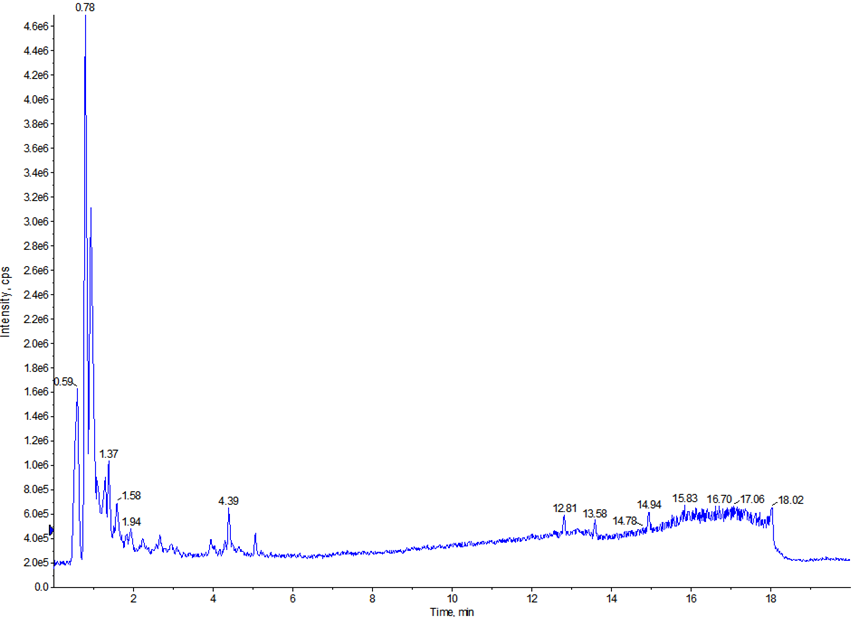


Fig. S1. Total ion chromatogram of AMEE acquired in the negative ion mode.


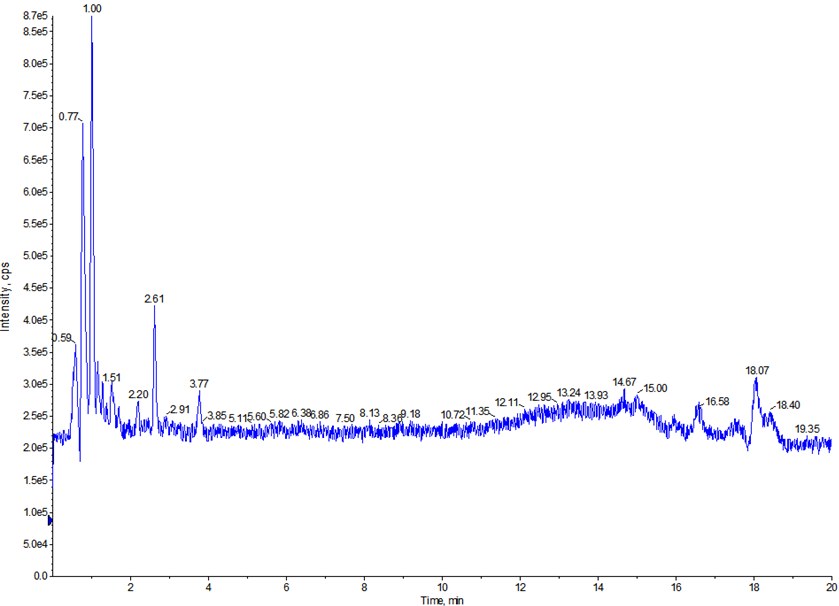


Fig. S2. Total ion chromatogram of AMEE acquired in the positive ion mode.
