# Supplementary material for: Anaphalis margaritacea ethanol extract exhibits potent anti-Trichinella spiralis activity via mitochondrial dysfunction and host tissue protection
Source: Int J Parasitol Drugs Drug Resist. 2026 May 18;31:100650. doi: 10.1016/j.ijpddr.2026.100650 (PMC13253213; doi:10.1016/j.ijpddr.2026.100650)
Supplement: Multimedia component 2 [file mmc2.docx]

Table S1. Extraction yields of water and 80% ethanol extracts from 30 medicinal plants used in this study

| No. | **Medicinal plants** | **Aqueous extract rate (%)** | **80% Ethanol extract rate (%)** |
| --- | --- | --- | --- |
| 1 | *Adiantum capillus-veneris* | 12.30% | 9.60% |
| 2 | *Agrimonia pilosa* | 16% | 13.50% |
| 3 | *Anaphalis margaritacea* | 12.90% | 9.50% |
| 4 | *Anemone hupehensis* | 14.30% | 15.70% |
| 5 | *Anemone tomentosa* | 8.50% | 12.30% |
| 6 | *Buddleja asiatica* | 10.10% | 6% |
| 7 | *Blumea balsamifera* | 13.60% | 10.80% |
| 8 | *Chelidonium majus* | 7.20% | 11.40% |
| 9 | *Cinnamomum camphora* | 3.50% | 4.10% |
| 10 | *Dictamnus dasycarpus* | 5.20% | 9.50% |
| 11 | *Eucalyptus globulus* | 3.30% | 3% |
| 12 | *Gelsemium elegans* | 14.20% | 15.80% |
| 13 | *Houttuynia cordata* | 3.50% | 6.40% |
| 14 | *Kalimeris indica* | 17.40% | 9.70% |
| 15 | *Leontopodium leontopodioides* | 12.60% | 13.90% |
| 16 | *Melia azedarach* | 12.80% | 10.30% |
| 17 | *Mirabilis jalapa* | 5.50% | 8.70% |
| 18 | *Phryma leptostachya subsp. asiatica* | 11.40% | 8% |
| 19 | *Phyllanthus emblica* | 14.30% | 10.50% |
| 20 | *Plumbago zeylanica* | 13.40% | 12.80% |
| 21 | *Polygonum hydropiper* | 4.20% | 6.10% |
| 22 | *Punica granatum* | 13.40% | 14.80% |
| 23 | *Rhamnus utilis* | 23.20% | 15% |
| 24 | *Sargentodoxa cuneata* | 6.90% | 8.60% |
| 25 | *Semen juglandis* | 12.50% | 10% |
| 26 | *Stemona japonica* | 2% | 5.70% |
| 27 | *Taxus wallichiana* | 6.80% | 12.70% |
| 28 | *Vernonia anthelmintica* | 1.50% | 2% |
| 29 | *Viticis* fructus | 7.20% | 6.40% |
| 30 | *Vitex negundo* | 8.90% | 9.30% |

Data are expressed as extraction yield (%) relative to the weight of dried crude drug (w/w).

Table S2. *In vitro* screening of plant extracts at 500 μg/mL against *T. spiralis* muscle larvae.

| No. | Medicinal plants | 80% Ethanol extract(500 µg/mL) | | | Aqueous extract(500 µg/mL) | | |
| --- | --- | --- | --- | --- | --- | --- | --- |
|  |  | 24h | 48h | 72h | 24h | 48h | 72h |
| 1 | Adiantum capillus-veneris | - | - | - | - | - | - |
| 2 | Agrimonia pilosa | - | - | - | - | - | - |
| 3 | *Anaphalis margaritacea* | 83.46±6.87 | 91.26±9.25 | 100±0.00 | - | - | - |
| 4 | *Anemone hupehensis* | - | - | - | - | - | - |
| 5 | *Anemone tomentosa* | - | - | - | - | - | - |
| 6 | *Buddleja asiatica* | - | - | - | - | - | - |
| 7 | *Blumea balsamifera* | - | - | - | - | - | - |
| 8 | Chelidonium majus | - | - | - | - | - | - |
| 9 | *Cinnamomum camphora* | - | - | - | - | - | - |
| 10 | Dictamnus dasycarpus | - | - | - | - | - | - |
| 11 | Eucalyptus globulus | - | - | - | - | - | - |
| 12 | *Gelsemium elegans* | 2.48±0.74 | 47.12±6.37 | 70.57±8.92 | - | - | - |
| 13 | Houttuynia cordata | - | - | - | - | - | - |
| 14 | *Kalimeris indica* | - | - | - | - | - | - |
| 15 | *Leontopodium leontopodioides* | 8.63±2.57 | 70.80±6.81 | 100±0.00 | - | - | - |
| 16 | *Melia azedarach* | 52.95±7.60 | 85.36±8.29 | 100±0.00 | - | - | - |
| 17 | *Mirabilis jalapa* | - | 24.89±7.92 | 72.69±6.43 | - | - | - |
| 18 | *Phryma leptostachya subsp. asiatica* | - | - | - | - | - | - |
| 19 | *Phyllanthus emblica* | 34.29±5.67 | 96.83±9.33 | 100±0.00 | - | - | - |
| 20 | *Plumbago zeylanica* | 7.7±2.45 | 73.33±8.10 | 100±0.00 | - | - | - |
| 21 | Polygonum hydropiper | - | - | - | - | - | - |
| 22 | *Punica granatum* | - | - | - | 62.47±6.25 | 93.14±5.34 | 97.59±3.21 |
| 23 | Rhamnus utilis | - | - | - | - | - | - |
| 24 | *Sargentodoxa cuneata* | 5.89±1.36 | 53.75±6.93 | 90.48±8.76 | - | - | - |
| 25 | *Semen juglandis* | - | - | - | 11.49±1.69 | 62.05±5.22 | 68.74±4.82 |
| 26 | *Stemona japonica* | - | - | - | - | - | - |
| 27 | *Taxus wallichiana* | - | - | - | 4.63±3.56 | 45.31±6.37 | 74.65±5.29 |
| 28 | *Vernonia anthelmintica* | - | - | - | - | - | - |
| 29 | Viticis fructus | - | - | - | - | - | - |
| 30 | Vitex negundo | - | - | - | - | - | - |

Data are expressed as larval mortality (%) at 24, 48 and 72 h. “–”, no detectable effect.

Table S3. Differentially expressed proteins selected for validation in AMEE-treated T. spiralis muscle larvae.

| Gene name | Protein description | log2FC | P Value | Regulation |
| --- | --- | --- | --- | --- |
| *Dnase2* | Deoxyribonuclease-2-alpha | -1.879 | 0.000414 | down-regulated |
| *nas-8* | Metalloendopeptidase | -1.694 | 0.0262 | down-regulated |
| *WAP* | Whey acidic protein | -1.681 | 0.0173 | down-regulated |
| *Fcn1* | Ficolin-1 | -1.45 | 0.0082 | down-regulated |
| *TXNDC11* | Thioredoxin domain-containing protein 11 | -1.416 | 0.000418 | down-regulated |

Table S3. Differentially expressed proteins selected for validation in AMEE-treated *T. spiralis* muscle larvae. Proteins were selected from the proteomic dataset based on the magnitude of differential expression and their potential relevance to representative biological processes, including secretion-related functions, stress responses, or mitochondrial-associated changes, for subsequent transcriptional validation by qPCR.

Table S4. Primer sequences used for quantitative real-time PCR

| Target | Sequence |
| --- | --- |
| *IL-18* | F:ATTGATCAAAGTGCCAGTGAAC  R:TGTTCTTACAGGAGAGGGTAGA |
| *Mmp9* | F:AGGAGTCTGGATAAGTTGGGTCTA  R:GCACACCAGAGAACTCCTTATCC |
| *Sod2* | F:CTGAAGAGCGACCTGAGTTGTAA  R:GCCGCAAAGAGTCTACATGTCTA |
| *Nqo1* | F:AAGCTGCAGACCTGGTGATATTT  R:GTTGTCGTACATGGCAGCATATG |
| *TSL-1* | F:AAGGTCCCTGCTGTGAATGAAAT  R:GGACTCCAGCTCCACAGTAAATT |
| *β-Actin* | F:CTACCTCATGAAGATCCTGACC  R:CACAGCTTCTCTTTGATGTCAC |
| *Fcn-1* | F:ACATTCAAATTGCCACCGTAAG-  R:TTGAGAGAAAGTCGCGGTATG |
| *TXNDC11* | F:CTGCCTGTCAGAGTTGGTAATC  R:AGCTTGCTAAAGCGGATAG |
| *WAP* | F:GAAGCATTGCAGGGTTCATTAG  R:TGCTCCAGTATTGTTGGTCTC |
| *nas-8* | F:CAAAGCCATGTCCTGAGAGTAG  R:CAACCAACCGGTCATCAAATC |
| *Dnase-2* | F:AGATTTCCCTCCTGGCAATTTA  R:GGTGTAACGCCCACTGAATA |
| *β-Tubulin* | F:GTGCCAGAGCTTACTCAGCA  R:AAGATCGCAGCCACTGTCAA |
